# Involvement of Multiple Types of Dehydrins in the Freezing Response in Loquat (*Eriobotrya japonica*)

**DOI:** 10.1371/journal.pone.0087575

**Published:** 2014-01-31

**Authors:** Hongxia Xu, Yong Yang, Li Xie, Xiaoying Li, Chao Feng, Junwei Chen, Changjie Xu

**Affiliations:** 1 Laboratory of Fruit Quality Biology/The State Agriculture Ministry Laboratory of Horticultural Plant Growth, Development and Quality Improvement, Zhejiang University, Zijingang Campus, Hangzhou, Zhejiang, People’s Republic of China; 2 Institute of Horticulture, Zhejiang Academy of Agricultural Sciences, Hangzhou, Zhejiang, People’s Republic of China; 3 MOA and Zhejiang Provincial Key Laboratory of Plant Virology, Institute of Virology and Biotechnology, Zhejiang Academy of Agricultural Sciences, Hangzhou, Zhejiang, People’s Republic of China; National Taiwan University, Taiwan

## Abstract

Dehydrins (DHNs) are a family of plant proteins typically induced in response to stress conditions that cause cellular dehydration, such as low temperatures, high salinity, and drought. Loquat (*Eriobotrya japonica*) is a perennial fruit crop that blossoms during winter. Loquat fruitlets are frequently injured by freezing. To evaluate the role of the *EjDHNs* in freezing resistance in loquat fruitlets, two cultivars of loquat, the freezing-sensitive ‘Ninghaibai’ (FS-NHB) and the freezing-tolerant ‘Jiajiao’ (FT-JJ), were analyzed under induced freezing stress. Freezing stress led to obvious accumulation of reactive oxygen species and considerable lipid peroxidation in membranes during the treatment period. Both these phenomena were more pronounced in ‘FS-NHB’ than in ‘FS-JJ.’ Immunogold labeling of dehydrin protein was performed. DHN proteins were found to be concentrated mainly in the vicinity of the plasma membrane, and the density of the immunogold labeling was significantly higher after freezing treatment, especially in the more freezing-tolerant cultivar ‘FT-JJ.’ Seven DHNs, showing four different structure types, were obtained from loquat fruitlets and used to study the characteristics of different *EjDHN* proteins. These DHN proteins are all highly hydrophilic, but they differ significantly in size, ranging from 188 to 475 amino acids, and in biochemical properties, such as theoretical pI, aliphatic index, and instability index. Freezing treatment resulted in up-regulation of the expression levels of all seven *EjDHNs*, regardless of structure type. The accumulation of the transcripts of these *EjDHN* genes was much more pronounced in ‘FT-JJ’ than in ‘FS-NHB.’ Altogether, this study provides evidence that *EjDHNs* are involved in the cryoprotection of the plasma membrane during freeze-induced dehydration in loquat fruitlets.

## Introduction

Because freezing temperatures are a major environmental constraint limiting the growth, development, and distribution of many kinds of plants, the mechanisms underlying freezing injury have been the subject of frequent study. Freezing injury is usually caused by cellular dehydration, and the plasma membrane is the primary site of freezing injury [1]. However, plants employ multiple mechanisms to increase their tolerance to freezing temperatures, such as accumulation of compatible osmolytes (soluble sugars, glycine betaine, and proline) and increased levels of antioxidants and soluble proteins in cell cytoplasm [2]–[3].

A set of cold-induced proteins have also received particular attention. Among these, DHNs, also known as LEA II (late embryogenesis abundant) proteins have been evaluated. The accumulation of DHNs in plants may be induced by abscisic acid (ABA) or any environmental influence that causes dehydration of the cells, such as freezing or other low temperatures, heat, high salinity, or drought [4]–[6]. Every protein in this family contains at least one copy of a lysine-rich amino acid sequence called the K-segment, which is usually located near the carboxyl terminus. It has a consensus sequence, EKKGIMDKIKEKLPG [7], [8]. DHNs may also possess one or more Y-segments, which is located near the amino terminus and has a consensus sequence, (V/T) DEYGNP, a S-segment containing multiple serine residues, or both [7], [8].

It is proposed that DHNs can protect proteins and membranes from unfavorable structural changes caused by dehydration. The K-segments form a putative amphiphilic α-helix domain. This domain involves interactions among hydrophobic and hydrophilic DHNs. DHNs may bind to intracellular macromolecules, coating them with a cohesive layer of water and preventing their coagulation during desiccation [8]. Several studies have shown that the expression and accumulation of DHN play an important role in the acclimation of fruit trees to unfavorable temperatures. The expression of CuCOR19, a DHN detected in the leaves of *Citrus unshiu*, was found to be significantly up-regulated by cold stress (4°C) but this was not the case with plants exposed to either ABA treatment or NaCl stress [9]. Three DHNs, 65, 60, and 14 kDa in size, were found to be strongly induced by cold in the floral buds of blueberry bushes, and the levels of these proteins were correlated with levels of resistance to cold [10]. The transcripts of *MdDHN*, a DHN isolated from apple trees, are highly expressed in bark and bud tissues when the plant is dormant during mid-winter, but they are not expressed during early spring when cold hardiness is lost and the buds are growing [11]. Transgenic approaches have been used to illustrate the relationship between DHN accumulation and cold acclimation. Hara *et al.* found that over-expression of CuCOR19 could enhance cold tolerance in transgenic tobacco and prevent lipid peroxidation [12]. Chen *et al*. have recently shown that overproduction of a *Brassica napus* DHN (*BnCOR*25) in yeast significantly increases the rate of cell survival under cold stress conditions and that over-expression of *BnCOR*25 in *Arabidopsis* increases plant tolerance to cold stress [13].

Loquat (*Eriobotrya japonica* Lindl.) is an important subtropical fruit. It has been cultivated commercially worldwide, especially in China, Japan, northern India, the Mediterranean, Brazil, the United States, Australia, and South Africa [14]. In the southeast of China, the loquat blooms continuously from October to January, and its fruitlets grow at the coldest time of the year. However, loquat fruitlets are sensitive to freezing stress. A reduction or cessation of growth frequently takes place during the winter. When the temperature drops below –3°C, many fruitlets suffer freezing-induced injury and die. This dramatically reduces yield. However, little information regarding the mechanisms underlying freezing injury in loquat is available. For this reason, the study of the physiological, biochemical and molecular characteristics of freezing stress in loquat fruitlets is required.

More structural and functional studies of DHNs have been performed in herbaceous plants than in other types of plants. *Arabidopsis*, wheat, and barley are the most frequent subjects. Recently, the identification and characterization of DHNs from woody plants has been reported in some species, including poplars, apples, and grapevine [15]–[17]. Many DHNs have been shown to be associated with the regulation of freezing tolerance in plants, rendering study of the function of the loquat DHN family highly relevant. However, information about the characteristics of this gene family in the loquat is limited. In a previous study, two *EjDHNs* were obtained and their expression patterns under different sets of low-temperature treatment conditions were subjected to preliminary investigation [18]. In the present study, seven *EjDHNs* were obtained and their roles in freezing resistance were analyzed in two loquat cultivars known to have different levels of sensitivity to freezing. It has been suggested that *EjDHNs* play an important role in maintenance of the stability of the plasma membrane during freezing-induced dehydration. Abundance of the transcripts of *EjDHNs* was found to be correlated with freezing tolerance in both cultivars.

## Materials and Methods

### Plant material

Two loquat (*Eriobotrya japonica* Lindl.) cultivars, a freezing-sensitive cultivar ‘Ninghaibai’ (FS-NHB) and a freezing-tolerant cultivar ‘Jiajiao’ (FT-JJ), grown in the Base Orchard of the Zhejiang Academy of Agricultural Sciences (Haining, China), were subjected to freezing treatments. *In vitro* branches bearing young fruit at 40 days after full bloom (DAFB) were collected from fields before the first cold spell and placed in a growth chamber (Sanyo, Japan) which was maintained at –3°C with a 12 h light/12 h dark photoperiod. Three biological replicates of fruit samples were collected at 0, 2, 4, 8, 12, and 24 h after the treatment. Untreated fruit (harvested at 0 h) served as controls. All collected samples were frozen in liquid nitrogen and stored at –80°C until analysis.

### Assessment of freezing tolerance

The freezing tolerance of the fruitlets was determined by the method of Sukumaran and Weiser [19]. *In vitro* normal growth loquat fruitlets (40 DAFB) were collected, then placed in stoppered culture tubes maintained in a low temperature bath (Masterline Model 2095, Forma Scientific) set at 0°C. Freezing was initiated by the addition of ice chips to each tube. After a 3 h equilibration period, the bath temperature was lowered in 2°C per hour until it reached –10°C. Samples were withdrawn at 0, –2, –4, –6, –8 and –10°C, and thawed overnight in a refrigerator at 4°C. Freezing damage was estimated by the electrolyte leakage test. Fruits were cut into small pieces (1 mm×1 mm×3 mm). Distilled water (30 ml) and 1.0 g samples were added to each tube, and the samples were shaken gently for 12 h. Conductivity of the resulting solution was measured using a conductance meter (DDS-320, Shanghai, China). Then the samples were boiled for 30 min, cooled at the room temperature for 3 h, and the final conductivity of the resulting solution was measured. Then the temperature at which 50% electrolyte leakage occurred (EL_50_) was determined and used to compare the freezing tolerance of the tested cultivars.

Analyses of malondialdehyde (MDA) content, hydrogen peroxide (H_2_O_2_) levels, and superoxide radical (O_2_^.−^ ) generation

The MDA content was determined using the thiobarbituric acid (TBA) reaction method as described by Dhindsa *et al*. [20]. H_2_O_2_ levels were determined as described by Velikova *et al*. [21]. The rate of O_2_^. −^ generation was measured by monitoring the amount of nitrite formed from hydroxylamine in the presence of O_2_^. −^ as described by Elstner and Heupel [22]. Soluble protein was monitored as described by Bradford [23].

### Measurement of enzyme activity

The fruit tissues were homogenized in 5 ml of 50 mM potassium phosphate buffer (pH 7.0) containing 4% (w:v) polyvinylpyrrolidon (Mr 25,000). The homogenate was centrifuged at 15,000 g for 20 min at 4°C. The supernatant was subjected to crude enzyme extraction.

The superoxide dismutase (SOD, EC 1.15.1.1) activity assay was based on the method described by Beauchamp and Fridovich [24]. The amount of enzyme capable of inhibiting 50% of the photochemical reduction of nitro-blue tetrazolium (NBT) at 560 nm here serves as one unit of enzyme activity. Three milliliters of reaction mixture contained 50 mM potassium phosphate buffer (pH 7.8), 0.1 mM EDTA, 13 mM methionine, 75 µM NBT and 16.7 µM riboflavin and 100 µl crude enzyme extract.

Ascorbate peroxidase (APX, EC 1.11.1.11) activity was determined as described by Nakano and Asada [25]. The assay mixture contained 50 mM potassium phosphate buffer (pH 7.0), 10 mM H_2_O_2_, and 0.5 mM each of AsA and enzyme extract. The reaction was initiated by addition of H_2_O_2_. One unit of enzyme activity was here defined as the amount of activity capable of causing a decrease of 0.01 at A290 per minute.

Catalase (CAT, EC 1.11.1.6) activity was determined by measuring the decomposition of H_2_O_2_ directly at 240 nm as described by Cheng *et al*. [26]. The reaction mixture contained 50 mM potassium phosphate buffer (pH 7.0), 10 mM H_2_O_2_, and 200 µl of enzyme extract in a 2 ml volume. One unit of enzyme activity was defined as the amount of activity capable of causing a decrease of 0.01 at A240 per minute.

### Immunogold labeling of DHN protein

Young fruits were cut into small pieces (1 mm×1 mm×3 mm) and fixed for 2 h at 4°C in 0.1 M phosphate buffer (pH 7.2), containing 3% (v/v) paraformaldehyde and 1% (v/v) glutaraldehyde. After washing with phosphate buffer, the pieces were dehydrated in an ethanol series and embedded in resin (Lowicryl K4M) [27]. Ultra-thin sections were placed on nickel grids and incubated first for 5 min in distilled water, then for 30 min with blocking solution containing 0.05 M PBS with 1% BSA, 0.02% PEG20000, 0.1 M NaCl, and 1% NaN_3_. Sections were then incubated on the primary antibody at a 1∶200 dilution in blocking solution for 2 h at 37°C. For the present experiment, anti-DHN antibody was purchased from Agrisera (Agrisera, Sweden). The immunogen of the antibody was a KLH-conjugated peptide sequence (K-segment) from the DHN C terminal, which is conserved across a wide range of plant species. After washing with distilled water, sections were incubated with colloidal gold (15 nm)-conjugated goat antiserum to rabbit immunoglobulins (secondary antibody) at a 1∶100 dilution in blocking solution for 2 h at 37°C. Subsequently, the sections were thoroughly washed with distilled water and counterstained with uranyl acetate and lead citrate and observed and photographed with an electron microscope (JEM-1200EX, JEOL, Japan).

### RNA extraction and cDNA synthesis

Total RNA was extracted from the frozen tissues using a modified version of the CTAB method [28]. After the removal of contaminating DNA using TURBO DNase (Ambion, USA), 1 µg of RNA was used for cDNA synthesis with a PrimeScript 1^st^ Strand cDNA Synthesis Kit (Takara, China) according to the manufacturer’s protocol. Tenfold diluted cDNA was used as the template for real-time quantitative PCR (Q-PCR) analysis. Three different RNA isolations and cDNA synthesis were used as replicates for the Q-PCR.

### Gene isolation

Seven DHN genes were isolated from another study involving RNA-seq. In that study, a mixture RNA from various tissues, including fruits at different stages of development and ripening, was sequenced using the latest Illumina deep sequencing technique. Four of the seven DHN genes were full-length sequences in the database, with complete start and stop codes, and full-length sequences of the other three DHN genes were obtained using RACE with a SMART RACE cDNA Amplification Kit (Clontech, USA), according to the manufacturer’s recommendations. The details of the primers employed for partial sequence and RACE amplification are given in Table S1 and Table S2. Full-length ORF was performed using the primers listed in Table S3. PCR product re-sequencing confirmation was also performed. cDNA from young fruit treated with –3°C for 24 h served as a template.

### Multiple sequence alignment, gene structure construction, and phylogenetic analysis

The protein MW (molecular weight), pI (isoelectric point), aliphatic index, instability index, and GRAVY (grand average of hydropathy) of the seven isolated *DHN* genes were predicted using the ProtParam program (http://au.expasy.org/tools/protparam.html) based on their amino acid compositions. The deduced amino acid sequences were aligned using ClustalX. A phylogenetic tree was generated using the neighbor-joining method with 1,000 bootstrap replicates. Multiple alignments of full-length DHN protein sequences from loquat, apple [16], barley [29]–[31], and *Arabidopsis* [32] were performed using the MEGA5 program [33].

### Gene expression analysis

The transcription levels of seven *DHN* genes were measured using Q-PCR with primers (Table 1) designed according to the obtained sequences (Figure S1) using Primer Premier 5 (Premier Biosoft International). The specificity of Q-PCR primers was determined by examining the melting peaks and dissociation curves. All Q-PCR products were cloned and re-sequenced to confirm that the primers were specific to the target genes.

**Table 1:** Primers used for Q-PCR amplification.

Gene	GenBank accession number	Forward primers(5′-3′)	Reverse primer(5′-3′)	Product size (bp)
*EjDHN1*	FJ472835	CCCGGCGGAAACCACTAGTGATATA	TGTATTAGCCGCACCAGAGCTGATC	141
*EjDHN2*	FJ472836	CTCCTCCCTGTGATGGGTGGTTTAT	GTCCTCCCAAACCAAAGAGAACCCT	111
*EjDHN3*	KF277187	CTGGTGTATAATAAGGGAGCGTCTG	CTGCTCTCAGAAATTAGCGCACAC	163
*EjDHN4*	KF277188	TCAGAACCAACACGGTGCAACACGC	CGTACCCGGTTGTGGCGGTACAGAA	107
*EjDHN5*	KF277189	GGAGAAGCCAGCTTCTTATCAGGAG	TGATGTGTACTGATCAGGAGCCGGT	109
*EjDHN6*	KF277190	CAATATGACACAACGCCCCAAGAC	CAGTACCGGTCTGGGCATTCGATGA	316
*EjDHN7*	KF277191	CGCACCGAGTAGATCACCATCCCGT	ACACCAGTGCGCAACGTGGATCACC	131
*Actin*	JN004223	GGATTTGCTGGTGATGATGC	CCGTGCTCAATGGGATACTT	172

The PCR mixture (10 µl total volume) contained 5.0 µl of SYBR^®^ Premix Ex Taq II (Takara), 0.4 µl of each primer (10 µM), 1.0 µl of diluted cDNA, and 3.2 µl of RNase-free water. PCR was performed on a LightCycler 480 instrument (Roche), initiated by 95°C for 30 s, then followed by 40 cycles of 95°C for 5 s, and 60°C for 20 s, and completed with a melting curve analysis program. No-template controls and melting curve analyses were included in every reaction. The level of actin expression was used to normalize the mRNA levels for each sample, and abundance was expressed in multiples of actin mRNA levels. The relative expression of EjDHNs was expressed as 2^− (Ct, Target − Ct, Actin)^ [34].

### Statistical analysis

Origin 7.0 (Microcal Software Inc.) was used to prepare the figures. All measurements were performed in three biological replicates and the results were expressed as mean values ± SE. The SPSS system (SYSTAT Version 11.5) was used for statistical analysis of the data. The significance of each variable among different cultivars was determined using the analysis of variance (ANOVA).

## Results

### Physiological changes in loquat fruitlets in response to freezing stress

**Figure 1:**
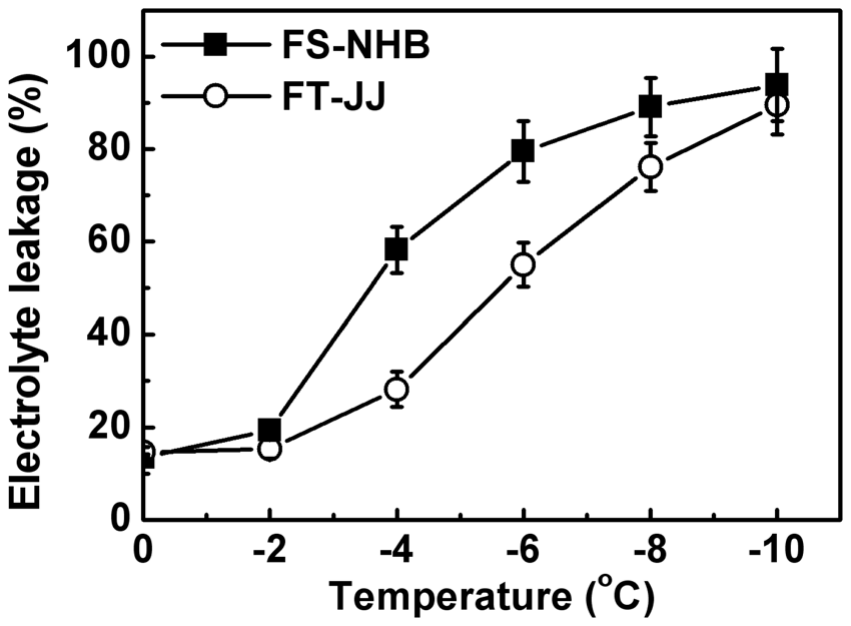
Leakage of electrolytes in loquat fruitlets from ‘FS-NHB’ and ‘FT-JJ’ when treated with temperatures below freezing. The data were presented as the mean ± SE (n  =  3).

**Figure 2:**
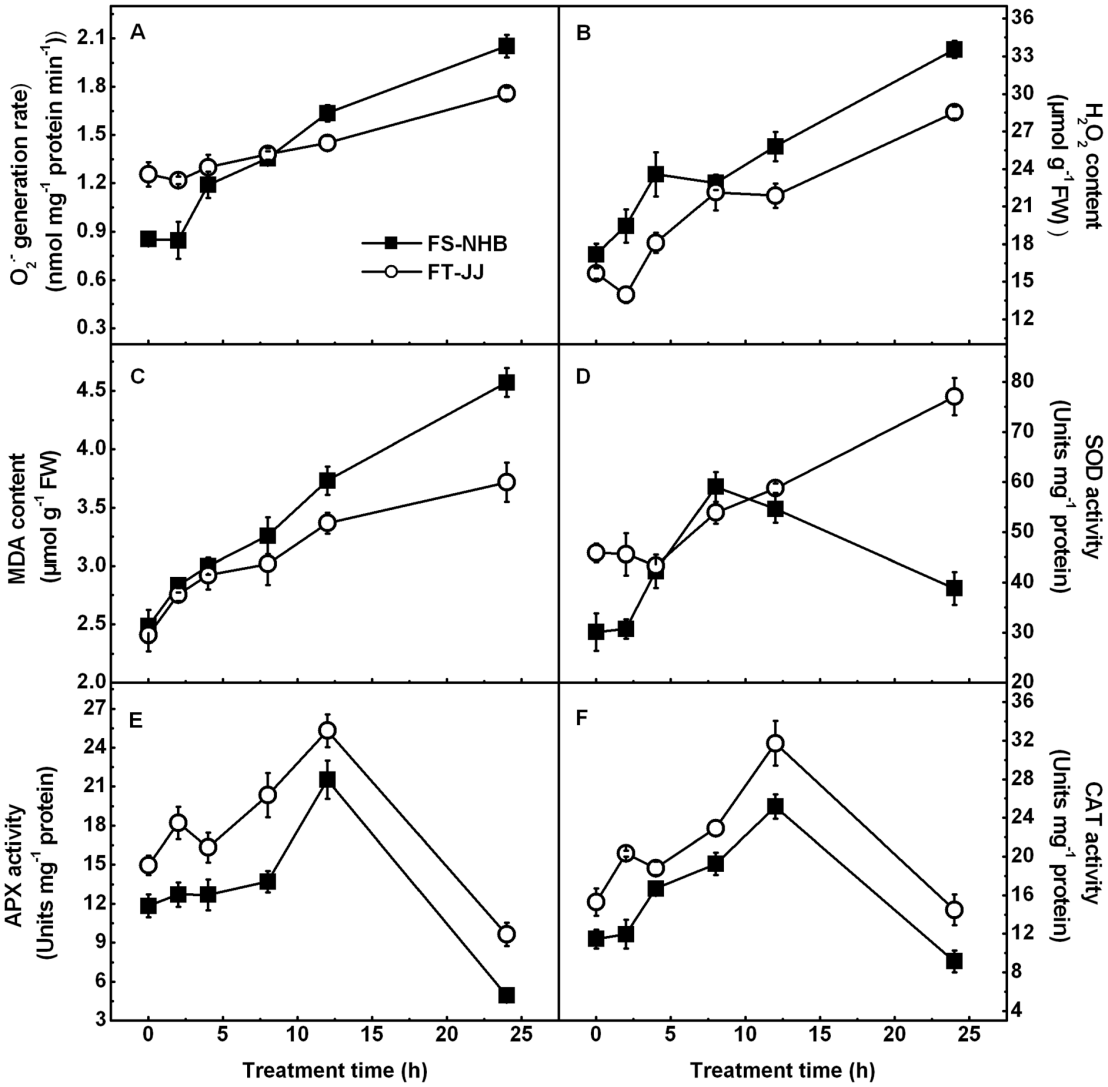
Physiological changes in ‘FS-NHB’ and ‘FT-JJ’ fruitlets during freezing treatment. A) O_2_^. −^ generation rate; B) H_2_O_2_ content; C) MDA content; D) SOD activity; E) APX activity; F) CAT activity. The data were presented as the mean ± SE (n  =  3).

Temperatures capable of causing 50% of electrolyte leakage (EL_50_) were approximately –3.7°C and –5.8°C, respectively, for ‘FS-NHB’ and ‘FT-JJ’ (Figure 1). This suggested that ‘FT-JJ’ was more tolerant to freezing stress than ‘FS-NHB’. Low-temperature stress leads to obvious physiological changes in loquats. MDA, H_2_O_2_ levels, and rate of O_2_^. −^ generation, and the activities of SOD, APX, and CAT were monitored (Figure 2). Many stresses, including low-temperature stress, always result in increases in the production of active oxygen species in plants. In the present experiment, the rate of O_2_^. −^ generation remained unchanged after 2 h of treatment but increased almost 141% and 40% after 24 h in ‘FS-NHB’ and ‘FT-JJ,’ respectively (Figure 2A). H_2_O_2_ content increased rapidly and reaching levels nearly a two-fold those observed at 0 h after 24 h in ‘FS-NHB.’ H_2_O_2_ content in ‘FT-JJ’ decreased slightly after 2 h, then increased rapidly during the prolonged treatment, finally peaking at 24 h (Figure 2B). MDA is believed to be the final product of lipid peroxidation in the plant cell membrane. It is an important indicator of membrane system injury and disruption of cellular metabolism [35]. Here, MDA content increased rapidly as freezing treatment continued, and the observed levels were increased by 84% and 54% after 24 h of stress treatment, in ‘FS-NHB’ and ‘FT-JJ,’ respectively (Figure 2C). SOD, APX, and CAT are three key antioxidant enzymes. These are usually up-regulated in plants subjected to stress, in which they scavenge reactive oxygen species (ROS). In the present experiment, the activity of SOD remained roughly constant across the first 2 h of treatment, then increased rapidly, peaking at 8 h, but levels decreased dramatically as treatment continued past that (12 h and 24 h) in ‘FS-NHB.’ However, in ‘FT-JJ,’ the activity of SOD was significantly higher after 4 h, and remained high through 24 h (Figure 2D). The changes in the activity levels of APX and CAT were similar in both ‘FS-NHB’ and ‘FT-JJ.’ They all increased within 12 h of the onset of stress but decreased sharply after 12 h, finally reaching their lowest level at 24 h. The activity levels of APX and CAT were significantly higher in ‘FT-JJ’ than in ‘FS-NHB’ throughout the treatment (Figure 2E, 2F).

### Immunogold labeling of DHN protein in response to freezing stress

**Figure 3:**
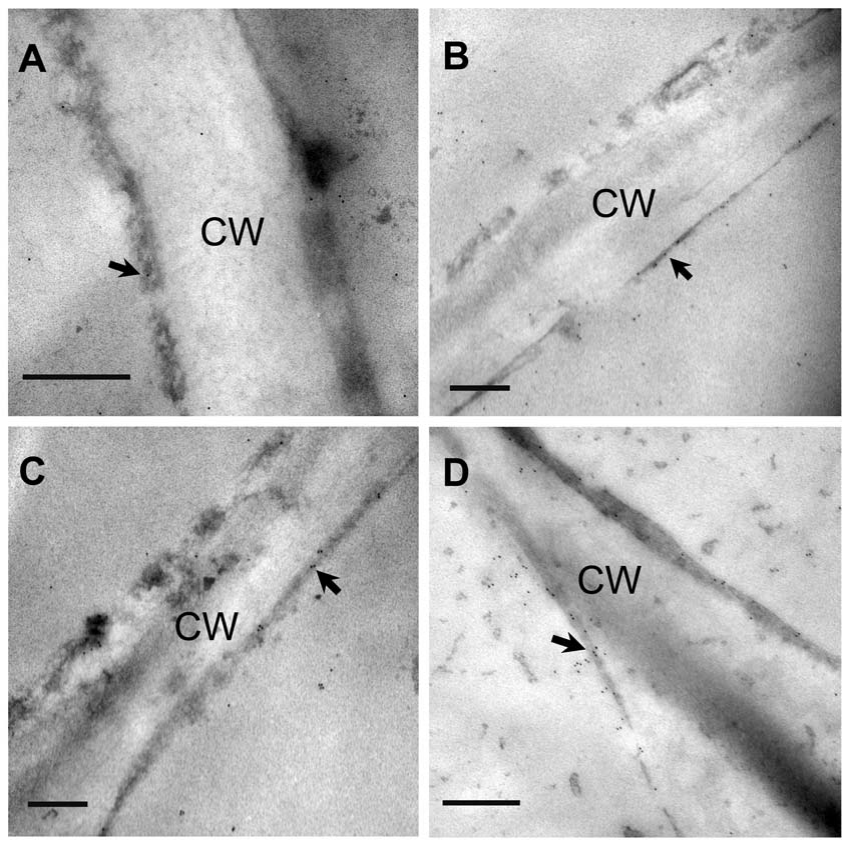
Immunogold labeling of dehydrin protein in loquat fruitlets. A) ‘FS-NHB’ before freezing stress; B) ‘FT-JJ’ before freezing stress; C) ‘FS-NHB’ after freezing stress; D) ‘FT-JJ’ after freezing stress. CW, cell wall; the arrow indicates the dense labeling on the plasma membrane; Bar  =  0.5 µm.

When ultrathin sections from fruit were incubated with the anti-DHN antibody and with gold-conjugated antiserum against rabbit immunoglobulins, deposition of gold particles over the cytoplasm and plasma membranes was observed (Figure 3). Quantitative evaluation was performed, and significantly more gold particles per µm^2^ along the plasma membrane were detected in ultrathin sections subjected to 24 h of freezing than those taken from fruit grown normally (Table 2). ‘FT-JJ’ showed more deposition of gold particles across the plasma membrane, lining the electron-opaque cell wall, than ‘FS-NHB’ for both normally grown and frozen fruitlets (Table 2).

**Table 2:** Immunogold labeling density of EjDHNs in fruitlets of ‘FS-NHB’ and ‘FT-JJ.’

Cultivar	FS-NHB		FT-JJ	
Treatment	CK	Freezing stress	CK	Freezing stress
Gold Particles per µm^2^ *	0.8±0.2 a	4.4±1.2 b	4.6±0.9 b	15.2±2.7 c

* Values are expressed as mean ± SE (n  =  5). Values followed by different letters are significantly different at *p*<0.05.

### Gene isolation and analysis

Seven putative genes were cloned using RT-PCR amplification. *EjDHN*1 and *EjDHN*2 were found to be same DHNs previously obtained by homology-based cloning with accession numbers FJ472835 and FJ472836 [18]. The other five DHNs have been deposited in GenBank with accession numbers KF277187– KF277191. There are five distinct structural types of DHNs known in plants: Y_n_SK_n_ DHNs, SK_n_ DHNs, K_n_ DHNs, Y_n_K_n_ DHNs, and K_n_S DHNs, where n indicates the number of repeats in each domain [9], [36]. In the present study, four types of DHNs were found, including Y_n_SK_n_ type of *EjDHN*1 (Y_2_SK_3_), SK_n_ type of *EjDHN*2 (SK_3_), *EjDHN*3 (SK_9_), *EjDHN*5 (SK_3_), *EjDHN*7 (SK_3_), K_n_ type of *EjDHN*4 (K_4_), and Y_n_K_n_ type of *EjDHN*6 (YK_3_) (Table 3). The proteins differed substantially in size, ranging from 188 (*EjDHN*1) to 475 (*EjDHN*3) amino acids in length. The predicted molecular weights of the seven deduced *EjDHN* proteins were between 19.9 kDa (*EjDHN*1) and 50.3 kDa (*EjDHN*3). All members of the DHN family were found to be highly hydrophilic, with a grand average of hydropathicity (GRAVY) values ranging from –1.244 (*EjDHN*4) to –1.698 (*EjDHN*2). The theoretical pIs ranged from 4.79 (*EjDHN*6) to 7.98 (*EjDHN*1), which showed that Y_n_SK_n_-type DHNs to possess a higher pI than K_n_, SK_n_, and Y_n_K_n_ type DHNs. The aliphatic index ranged from 18.53 (*EjDHN*6) to 41.79 (*EjDHN*2). The predicted results also showed that *EjDHN*2 and *EjDHN*5 were unstable (instability index > 40), while the other five DHN proteins were stable (instability index <40).

**Table 3:** Characteristics of loquat DHN protein sequence features.

Name	Type	AA	MW (kDa)	pI	Aliphatic index	Instability index	GRAVY
EjDHN1	Y_2_SK_3_	188	19.9	7.98	33.30	25.44	–1.269
EjDHN2	SK_3_	273	31.6	5.28	41.79	54.03	–1.698
EjDHN3	SK_9_	475	50.3	7.24	34.57	23.24	–1.288
EjDHN4	K_4_	200	21.5	6.45	30.35	1.18	–1.244
EjDHN5	SK_3_	284	32.8	5.17	41.23	57.94	–1.646
EjDHN6	YK_3_	190	20.2	4.79	18.53	16.53	–1.322
EjDHN7	SK_3_	193	20.3	6.12	32.38	24.89	–1.324

**Figure 4:**
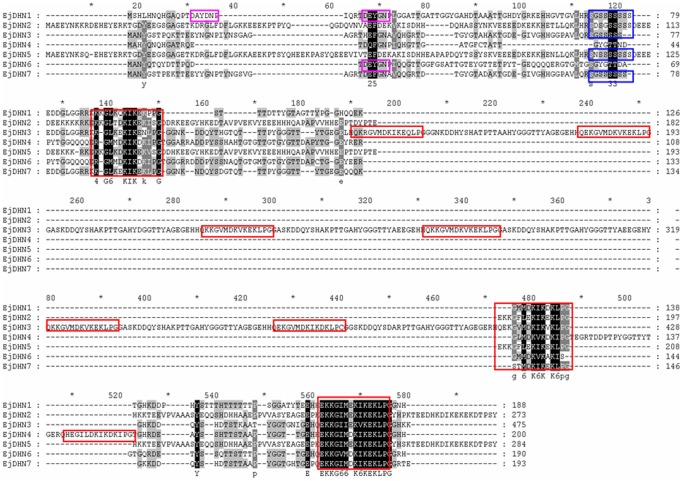
Alignment of multiple EjDHN amino acid sequences. Conserved amino acid sequences are indicated by pink boxes for the Y-segment; blue boxes for the S-segment; and red boxes for the other Y-segment.

Alignments of full-length deduced proteins showed conserved motifs, including Y-, K-, and S-motifs. These motifs were found to be highly conserved through the loquat DHN family. The present results also showed that the remaining regions other than EjDHN2 and EjDHN5 displayed relatively low amino acid identity across the DHNs (Figure 4).

**Figure 5:**
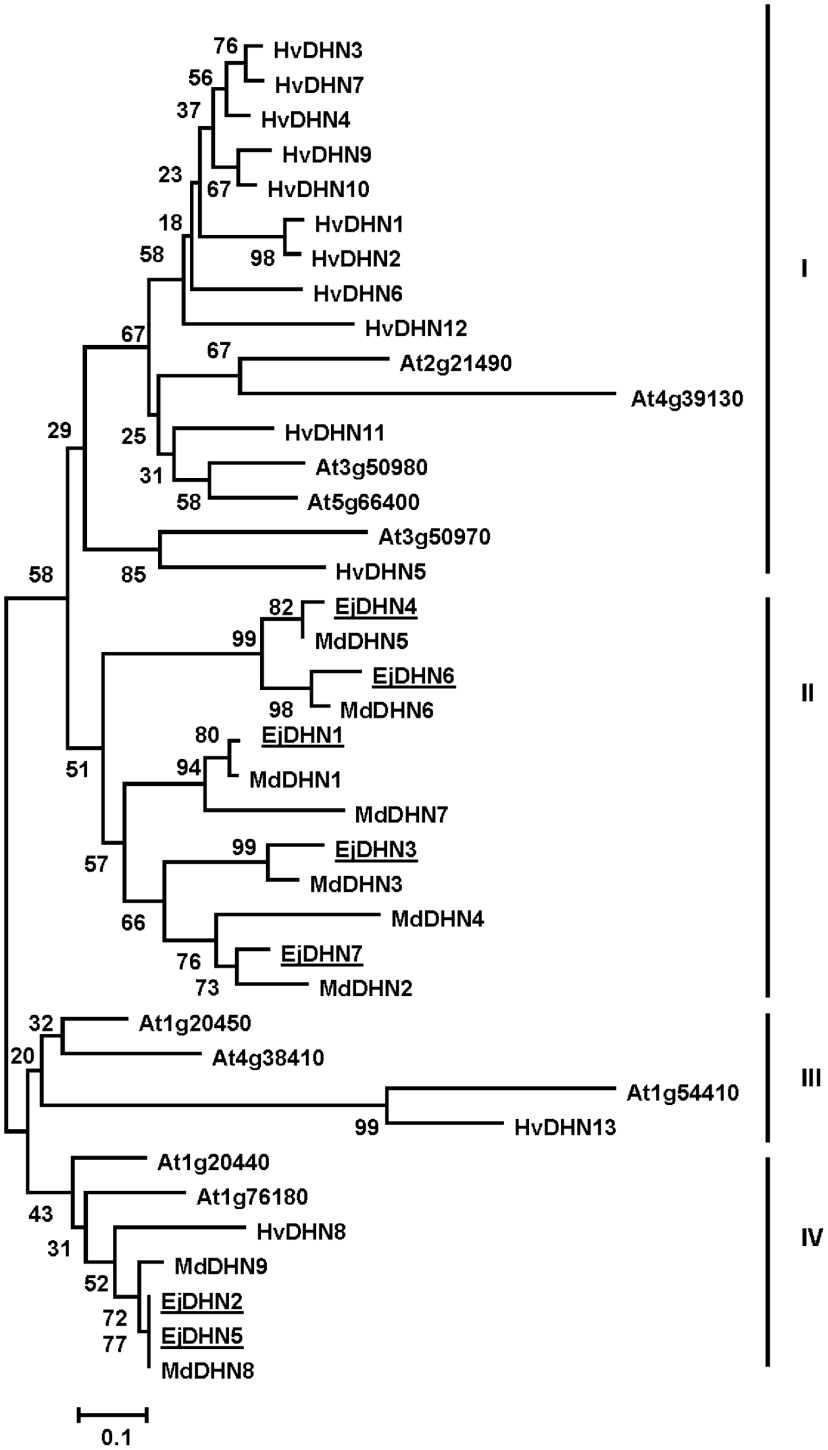
Phylogenetic analysis of dehydrin proteins from loquat, apple, *Arabidopsis thaliana*, and barley. An unrooted tree was generated using the MEGA5.0 program and neighbor-joining method. Proteins were arranged into classes I, II, III, and IV based on sequence similarities. GenBank accession numbers are as follows: seven loquat (*Eriobotrya japonica*; Ej) dehydrins including EjDHN1 (FJ472835), EjDHN2 (FJ472836), EjDHN3 (KF277187), EjDHN4 (KF277188), EjDHN5 (KF277189), EjDHN6 (KF277190), and EjDHN7 (KF277191); nine apple (*Malus domestica*, Md) dehydrins including MdDHN1 (JQ649456), MdDHN2 (JQ649457), MdDHN3 (JQ649458), MdDHN4 (JQ649459), MdDHN5 (JQ649460), MdDHN6 (JQ649461), MdDHN7 (JQ649462), MdDHN8 (JQ649463), and MdDHN9 (JQ649464); ten *Arabidopsis thaliana* (At) dehydrins including At1g20440 (AY11 4699), At1g20450 (AF360351), At1g54410 (NM_104319), At1g 76180 (AF339722), At2g21490 (BT000900), At3g50970 (NM_114957), At3g50980 (NM_114958), At4g38410 (NM_120003), At4g39130 (NM_120073), and At5g66400 (AY093779); and thirteen barley (*Hordeum vulgare*, Hv) dehydrins including HvDHN1 (AF043087), HvDHN2 (AF181452), HvDHN3 (AF181453), HvDHN4 (AF181454), HvDHN5 (AF181455), HvDHN6 (AF181456), HvDHN7 (AF181457), HvDHN8 (AF181458), HvDHN9 (AF181459), HvDHN10 (AF181460), HvDHN11 (AF043086), HvDHN12 (AF155129), and HvDHN13 (AY681974).

To study the phylogenetic relationships among loquats, apples, *Arabidopsis*, and barley, an un-rooted tree of DHN proteins was generated using the neighbor-joining method and MEGA5.0. This tree was based on the deduced protein sequences of seven EjDHNs, nine MdDHNs, ten AtDHNs, and thirteen HvDHNs. The DHNs could be divided into four groups according to the phylogenetic results (Figure 5). Five EjDHNs were placed in Group II, which also had seven MdDHN proteins but no AtDHNs or HvDHNs. Group IV included the other two EjDHNs, one HvDHN, two MdDHNs, and two AtDHNs. However, both group I and group III included only HvDHN and AtDHN proteins, with no EjDHNs or MdDHNs. This phylogenetic analysis demonstrated that all of our loquat DHN genes were more closely related to apple genes than similar genes in other species.

### Expression patterns of *EjDHN* genes in response to freezing stress

**Figure 6:**
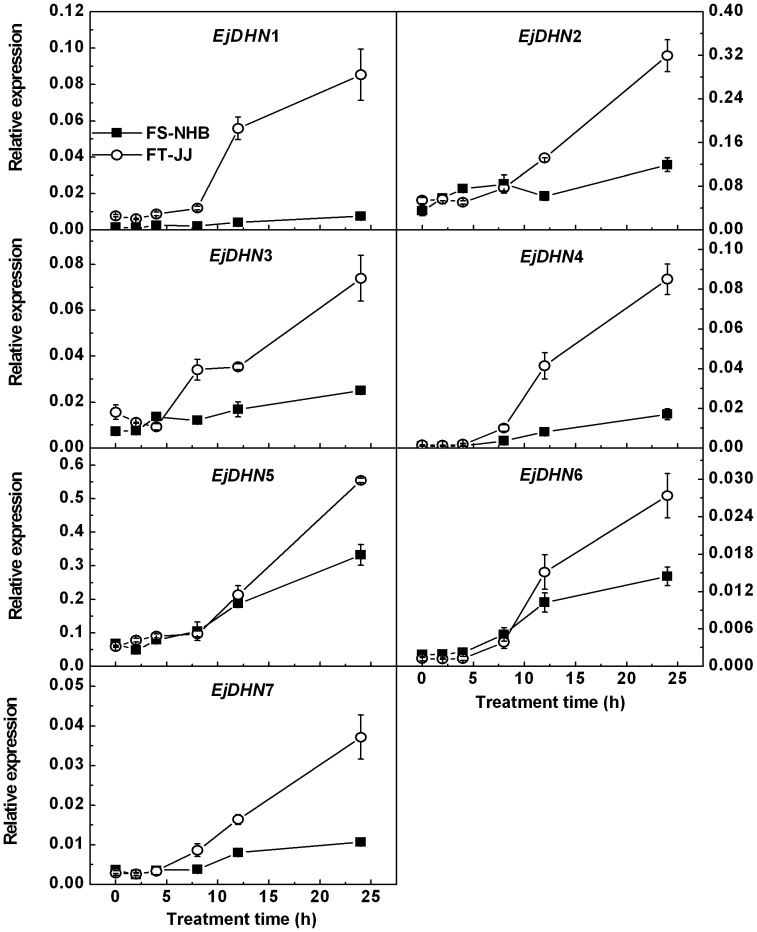
Expression patterns of *EjDHN* genes in ‘FS-NHB’ and ‘FT-JJ’ fruitlets during freezing treatment. The level of actin expression was used to normalize the mRNA levels in each sample, and mRNA levels produced by Q-PCR are expressed relative to actin levels. The data were presented as means mean ± SE (n  =  3).

The possibility that differences in the freezing tolerance of ‘FS-NHB’ and ‘FT-JJ’ fruits could be related to the expression of DHN genes was investigated. Real-time RT-PCR analyses were used to examine the expression of *EjDHN*s in response to 0, 2, 4, 8, 12, and 24 h of -3°C treatment (Figure 6). The responses of the *DHN* genes to low-temperature treatment in ‘FS-NHB’ were different from those in ‘FT-JJ.’ The concentration of the transcript of *EjDHN*1 remained constant in ‘FS-NHB’ fruit during throughout the treatment process, but in ‘FT-JJ,’ it increased significantly after 8 h of treatment and peaked at 24 h after treatment. *EjDHN*2 was unresponsive to treatment before 12 h in ‘FS-NHB,’ but it increased afterward, and peaking at 24 h. *EjDHN*2 expression was found to be dramatically induced by low-temperature stress after 4 h in ‘FT-JJ,’ and it increased 6-fold at 24 h. The freezing showed no significant effect on *EjDHN*3 expression in ‘FS-NHB,’ but it was markedly increased in ‘FT-JJ.’ Like the *EjDHN*1, *EjDHN*2, and *EjDHN*3 genes, the *EjDHN*4 gene exhibited a differential response to freezing treatment. Expression increased 18.4-fold in ‘FS-NHB’ and 80.6-fold in ‘FT-JJ’ after 24 h of treatment. Abundance of the *EjDHN*5 transcript was higher than that of the other six DHNs in both ‘FS-NHB’ and ‘FT-JJ.’ It also increased markedly after 8 h of treatment and induced by 4.9-fold in ‘FS-NHB’ and 8.3-fold in ‘FT-JJ’ at 24 h. The expression of *EjDHN*6 and *EjDHN*7 was also induced by freezing stress, as observed at 8, 12, and 24 h. In ‘FT-JJ’ fruit, the abundance of the transcripts of *EjDHN*6 increased by 22.1-fold and that of *EjDHN*7 by 12.9-fold in at 24 h, but in ‘FS-NHB,’ levels remained relative stable throughout this stress period.

## Discussion

### Gene types versus transcription levels of *EjDHN*s in response to freezing stress

It has been suggested that different types of DHN proteins are involved in responses to various growth conditions. Basic and neutral dehydrins, such as Y_n_SK_n_ dehydrins, are usually responsive to drought and to ABA but not to cold, but acidic DHNs, such as K_n_, SK_n_, and YK_n_ DHNs, are preferentially induced by low temperatures [36]. It has been confirmed that K_n_ DHNs are directly involved in cold acclimation processes [29], [37]. *EjDHN*4, a K_n_ type gene, was found to be the most sensitive to low-temperature of any *DHN*. The transcript abundance of *EjDHN*4 increased dramatically after 8 h of freezing treatment and the magnitude of the increase that took place during 24 h of treatment was greater than that of other DHNs. It was also observed that the levels transcription of *EjDHN*5 were markedly higher than those of the other *DHN*s, which was followed by *EjDHN*2 (Figure 6). Therefore, it is here suggested that *EjDHN*5 and *EjDHN*2 may be more important than other *EjDHN*s with respect to resistance to freezing temperatures among loquat fruitlets.

The phylogenetic tree shows that *EjDHN*2 and *EjDHN*5 should be placed in group IV, which contains all SK_n_-type DHNs. The acidic dehydrin from barley, *HvDHN*8, has been shown to be actively expressed during low-temperature treatment [29]. Of the DHN genes, At1g76180 (encoding ERD14) and At1g20440 (encoding COR47) have been reported to be up-regulated under cold stress conditions [2], [38]. They are major cold-induced DHNs in *Arabidopsis*. The *EjDHN*2 and *EjDHN*5 sequences appeared to be highly homologous and to differ only in the presence of short indels. In this way, they were similar to *MdDHN*8 and *MdDHN*9 (Figure 4). However, unlike *MdDHN*8, which is induced by chilling stress, the concentrations of the *MdDHN*9 transcript have been found to remain in chilled apple seedlings [16]. In the present study, all seven isolated *EjDHN*s were up-regulated under low-temperature stress conditions, although *EjDHN*1 and *EjDHN*3 are basic DHNs. In apples, transcripts of basic DHNs MdDHN2 and MdDHN4 were dramatically increased when seedlings exposed to 4°C [16].

As mentioned above, the seven *EjDHN*s were obtained from loquat transcriptome data. These were obtained from a mixture of all tissues, including stems, leaves, buds, flowers, roots, and fruits. The transcriptome data contained 11 UniGenes of DHN. However, four of them could not be isolated from loquat fruitlets. It can be possible that these four *EjDHN*s may express in tissues other than fruitlets, and their expression patterns in response to freezing stress are still unknown.

### Expression levels of *EjDHN*s versus freezing tolerance of different loquat genotypes

Decreases in membrane fluidity, as a result of peroxidation, may be one cause of the cold-stress-induced membrane injury [39]. Thompson *et al*. found lipid peroxidation in plant membranes to be caused primarily by free-radical attacks [40]. The antioxidant systems found in plants are very complex in plants. There are numerous antioxidants and some special proteins involved. The physiological changes in loquats under low-temperature stress conditions included dramatically elevated levels of antioxidant enzyme activity could not scavenge the ROS effectively, and the membrane systems were then damaged by ROS. Membrane system injury was more pronounced in ‘FS-NHB’ than in ‘FT-JJ’ (Figure 2). This may be partially attributable to the lower levels of activity of its antioxidant enzymes.

It has been frequently shown that DHNs can alleviate oxidative damage in stressed plants by scavenging hydroxyl and peroxyl radicals or binding metals [41]–[43]. Hara *et al*. also proved that Gly, His, and Lys, which are major residues in many DHNs, may be targets of these radicals [42]. For loquats, the Gly, His, and Lys residues in DHNs accounted for an average of 14.4%, 5.3%, and 11.0% of all amino acids, respectively (Table S4). This shows that the EjDHNs might also be able to alleviate oxidative damage.

Both Q-PCR and immunoelectron microscopic analyses provide evidence that the expression and accumulation of DHN proteins during freezing treatment are correlated with the different levels of tolerance to freezing conditions observed in loquat cultivars. In the present study, *EjDHN* genes were found to be more sensitive to freezing stress, and the level of expression was much higher in ‘FT-JJ’ than in ‘FS-NHB’ (Figure 6). Data from immunoelectron microscopic analyses also indicated that ‘FT-JJ’ has more gold particles deposited on the plasma membrane than ‘FS-NHB’ (Figure 3B, 3D). These results were consistent with those of previous studies. Danyluk *et al.* found a positive correlation between the accumulation of *Wcor410* transcripts and the capacity of different wheat cultivars to tolerate freezing conditions [44]. Fernandez *et al*. isolated three cold-acclimation-responsive DHN genes from blue gum, and they found the level of transcription of these three DHN genes to be higher in a freezing-resistant genotype compared to a sensitive genotype [45]. The severe membrane damage observed in ‘FS-NHB’ plants after freezing treatment may be partially ascribed to its lower ability to accumulate DHN proteins.

### Subcellular localization versus cryoprotection of the plasma membrane of EjDHNs

DHNs are located in the cytoplasm, plasma membrane, nucleus, and mitochondria [7], [12], [46], [47]. Because cell membranes are the primary sites of freezing injury, changes in membrane behavior in response to low-temperature stress are critical to the development of freezing tolerance. It is believed that DHNs can interact with cell endo membranaceous systems and partially unfolded proteins *via* the hydrophobic side of the K-segment and therefore protect them from unfavorable changes during dehydration [46], [48]. Eriksson *et al*. had demonstrated that K-segments were implicated in membrane binding [49]. They found that K-segments could lower the temperature of the main lipid phase transition. In the present study, through immunoelectron microscopic analyses, it was observed that DHN proteins are concentrated mainly near the plasma membrane after forest treatment, and the density of the immunogold labeling was significantly higher than in controls (Figure 3, Table 2). Similar results were observed by Danyluk *et al*. [46]. They found that WCOR410 proteins accumulated near the plasma membranes of cells in the sensitive vascular transition area, where freeze-induced dehydration is most likely to be severe. The seven DHN proteins obtained in the present study are all hydrophilic, as indicated by their GRAVY values, which are ≤ –1.244 (Table 3). Results concerning the properties, expression patterns, and localization of these proteins support that the *EjDHN*s might be involved in preventing the destabilization of the plasma membrane that occurs during freezing-induced dehydration.

## Conclusion

Plants of ‘FS-NHB,’ a freezing sensitive loquat cultivar, suffered much more serious membrane system injuries and disruption of cellular metabolism than ‘FT-JJ,’ a freezing-tolerant cultivar, when subjected to freezing temperatures. Seven members of the loquat DHN family were identified and characterized. The amount of DHN proteins accumulated, as well as the abundance of *EjDHN* transcripts, showed quantitative differences between these two contrasting cultivars. It can be concluded that the EjDHN protein family is involved in the cryoprotection of the plasma membrane during freezing-induced dehydration of loquat fruitlets.

## Supporting Information

Figure S1 **Alignment of multiple** ***EjDHN*** **nucleotide sequences.** Specific regions used for developing Q-PCR primers of each *EjDHN* were underlined. (XLS).
(TIF)

Table S1 **Primers for partial sequence amplification.**
(DOC)

Table S2 **Primers used for 3′ and 5′ race.**
(DOC)

Table S3 **Primers used for full ORF amplification.**
(DOC)

Table S4 **Amino acid composition of EjDHNs.**
(DOC)
