# Supplementary figures and images for: Involvement of Multiple Types of Dehydrins in the Freezing Response in Loquat (Eriobotrya japonica)
Source: PLoS One. 2014 Jan 31;9(1):e87575. doi: 10.1371/journal.pone.0087575 (PMC3909202; doi:10.1371/journal.pone.0087575)

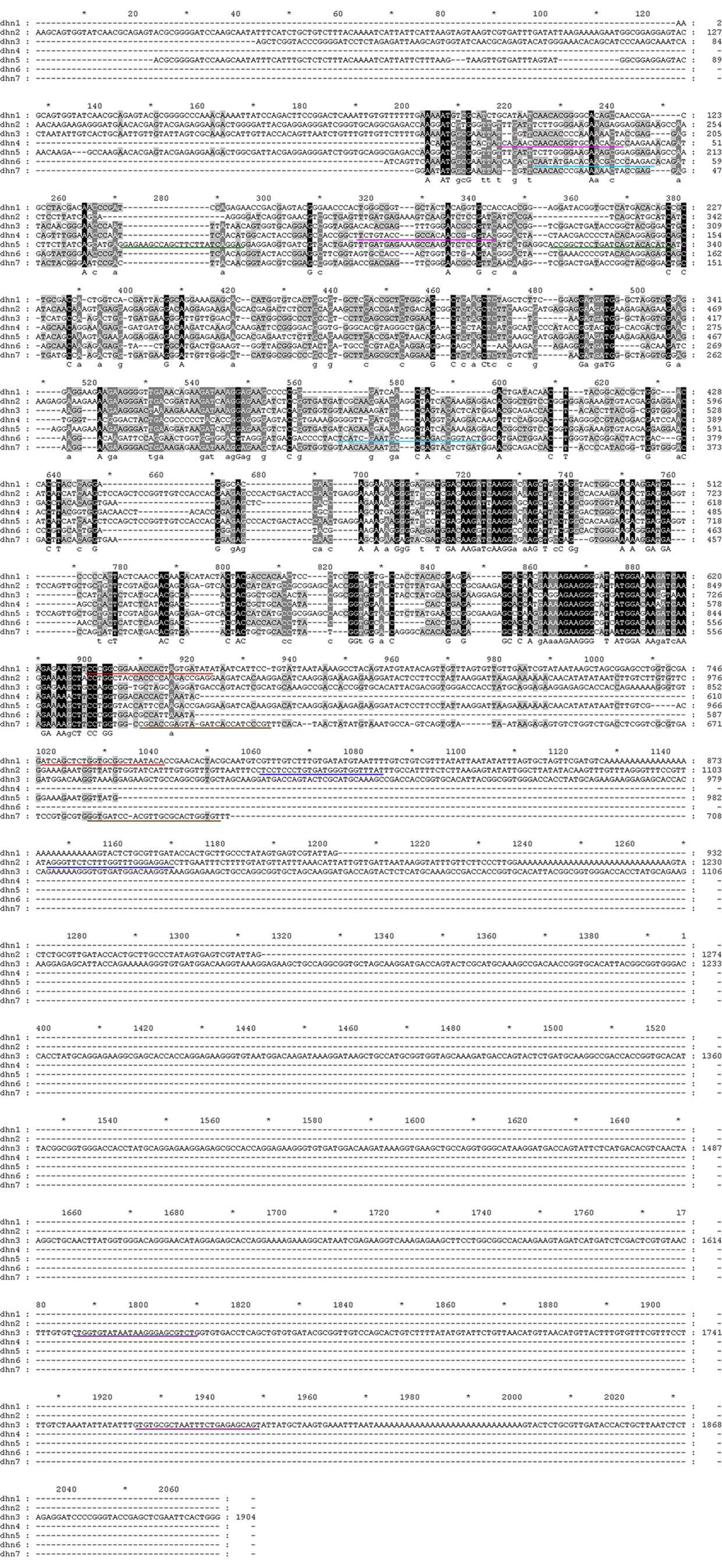

Supplement: Figure S1 — Alignment of multiple EjDHN nucleotide sequences. Specific regions used for developing Q-PCR primers of each EjDHN were underlined. (XLS). (TIF) [file pone.0087575.s001.tif]
