# Supplementary material for: Involvement of Multiple Types of Dehydrins in the Freezing Response in Loquat (Eriobotrya japonica)
Source: PLoS One. 2014 Jan 31;9(1):e87575. doi: 10.1371/journal.pone.0087575 (PMC3909202; doi:10.1371/journal.pone.0087575)
Supplement: Table S1 — Primers for partial sequence amplification. (DOC) [file pone.0087575.s002.doc]

**Table S1.** Primers for partial sequence amplification.

| Primer name | Primer sequence (5′–3′) |
| --- | --- |
| *EjDHN1*-p-Forward | ACTGATGAGTATGGGAACC |
| *EjDHN1*-p-Reverse | ACCTGGCAGCTTGTCCTTGA |
| *EjDHN2*-p-Forward | GAAGGTCTCCGGTGATGATCACAAG |
| *EjDHN2*-p-Reverse | TCTTGGGGTGGTAGCCAGGTAGCTT |
| *EjDHN3*-p-Forward | CCTTGCTGGTGCGAATCAGTTGTCT |
| *EjDHN3*-p-Reverse | TGGTGGTGCTCTCCTTCTCCTGCAT |
