# Supplementary material for: Involvement of Multiple Types of Dehydrins in the Freezing Response in Loquat (Eriobotrya japonica)
Source: PLoS One. 2014 Jan 31;9(1):e87575. doi: 10.1371/journal.pone.0087575 (PMC3909202; doi:10.1371/journal.pone.0087575)
Supplement: Table S2 — Primers used for 3′ and 5′ race. (DOC) [file pone.0087575.s003.doc]

**Table S2.** Primers used for 3′ and 5′ race.

| Primer name | Primer sequence (5′–3′) |
| --- | --- |
| *EjDHN1*-3′-GPS1 | GGGCACCAACAGGAAAAGGGGATG |
| *EjDHN2*-3′-GPS1 | GGAGGTTCCAGTTGCTGCTGCTTCG |
| *EjDHN3*-3′-GPS1 | CAGGTGAAGGAGAGCTCCACCAGAA |
| *EjDHN1*-5′-GPS2-1 | TCCCCTTTTCCTGTTGGTGCCCTC |
| *EjDHN1*-5′-GPS2-2 | GAGCGGTGGAGCACGCCAGTGACA |
| *EjDHN2*-5′-GPS2-1 | ACGAAGCAGCAGCAACTGGAACCT |
| *EjDHN2*-5′-GPS2-2 | GTGGTGGACAACCGGAGCTGGAGC |
| *EjDHN3*-5′-GPS2-1 | GCTGGACAACCGCGTATCACACAGC |
| *EjDHN3*-5′-GPS2-2 | GCTCTCCTATGTTCCCTGTCCCACC |
| NUP | AAGCAGTGGTATCAACGCAGAGT |
| UPM | CTAATACGACTCACTATAGGGCAAG  CAGTGGTATCAACGCAGAGT |
