# Supplementary material for: Involvement of Multiple Types of Dehydrins in the Freezing Response in Loquat (Eriobotrya japonica)
Source: PLoS One. 2014 Jan 31;9(1):e87575. doi: 10.1371/journal.pone.0087575 (PMC3909202; doi:10.1371/journal.pone.0087575)
Supplement: Table S3 — Primers used for full ORF amplification. (DOC) [file pone.0087575.s004.doc]

**Table S3.** Primers used for full ORF amplification

| Genes | GenBank accession numbers | Primers | Sequences (5′–3′) |
| --- | --- | --- | --- |
| *EjDHN1* | FJ472835 | Forward | TTGAAAAATGTCGCATCTG |
|  |  | Reverse | CAACAACACTAAACAACTG |
| *EjDHN2* | FJ472836 | Forward | GAAAGTATGGCGGAGGAGTACAACA |
|  |  | Reverse | CCGTTAATTAATAGGAAGGAGTATCC |
| *EjDHN3* | KF277187 | Forward | AATGGCGAATTATGGTTCAACACCC |
|  |  | Reverse | CCAGACGCTCCCTTATTATACACCA |
| *EjDHN4* | KF277188 | Forward | ACAATGGCGCACTATCAGAACC |
|  |  | Reverse | GTATTTAGTGGTGTCCACCGGGAAG |
| *EjDHN5* | KF277189 | Forward | ACGCGGGGATCCAAGCAATATTTCA |
|  |  | Reverse | CATTCTTTCCGAACACAAGACAAGAT |
| *EjDHN6* | KF277190 | Forward | ATCAGTTCAAAATGGCGAATTATCA |
|  |  | Reverse | TATTTAATGGCGTCCACCGGGAAGC |
| *EjDHN7* | KF277191 | Forward | GGAATATGGCGAATTATGGTTCAAC |
|  |  | Reverse | AAACACCAGTGCGCAACGATGGATC |
