# Supplementary material for: Involvement of Multiple Types of Dehydrins in the Freezing Response in Loquat (Eriobotrya japonica)
Source: PLoS One. 2014 Jan 31;9(1):e87575. doi: 10.1371/journal.pone.0087575 (PMC3909202; doi:10.1371/journal.pone.0087575)
Supplement: Table S4 — Amino acid composition of EjDHNs. (DOC) [file pone.0087575.s005.doc]

**Table S4.** Amino acid composition of EjDHNs.

| Amino acid composition | EjDHN1 | EjDHN2 | EjDHN3 | EjDHN4 | EjDHN5 | EjDHN6 | EjDHN7 |
| --- | --- | --- | --- | --- | --- | --- | --- |
| Ala (A) | 5.3% | 5.1% | 6.7% | 5.5% | 6.0% | 3.2% | 4.1% |
| Arg (R) | 2.7% | 2.2% | 1.7% | 4.5% | 1.4% | 3.7% | 3.1% |
| Asn (N) | 2.1% | 1.1% | 1.9% | 1.5% | 1.1% | 1.6% | 3.1% |
| Asp (D) | 6.4% | 7.7% | 6.5% | 7.5% | 6.3% | 6.8% | 6.7% |
| Cys (C) | 0.0% | 0.0% | 0.2% | 0.5% | 0.0% | 0.5% | 0.0% |
| Gln (Q) | 3.2% | 2.9% | 4.2% | 5.0% | 2.8% | 8.9% | 5.2% |
| Glu (E) | 5.3% | 19.4% | 7.4% | 5.5% | 20.4% | 6.8% | 7.8% |
| Gly (G) | 16.0% | 5.9% | 18.7% | 17.5% | 4.9% | 18.9% | 19.2% |
| His (H) | 8.0% | 6.6% | 6.9% | 6.0% | 6.7% | 1.1% | 2.1% |
| Ile (I) | 2.7% | 2.6% | 1.9% | 4.5% | 2.5% | 2.6% | 2.6% |
| Leu (L) | 3.7% | 3.3% | 2.7% | 1.5% | 3.2% | 0.5% | 3.1% |
| Lys (K) | 9.6% | 16.5% | 12.0% | 7.0% | 15.8% | 5.8% | 10.4% |
| Met (M) | 2.1% | 0.7% | 1.7% | 2.5% | 0.7% | 4.2% | 1.6% |
| Phe (F) | 0.0% | 1.5% | 0.2% | 1.0% | 1.4% | 0.5% | 0.5% |
| Pro (P) | 5.3% | 5.5% | 4.0% | 5.5% | 5.6% | 4.7% | 4.7% |
| Ser (S) | 6.4% | 5.9% | 4.4% | 2.5% | 7.7% | 4.2% | 6.7% |
| Thr (T) | 16.0% | 4.0% | 9.5% | 16.0% | 3.9% | 18.4% | 11.9% |
| Trp (W) | 0.0% | 0.0% | 0.0% | 0.0% | 0.0% | 0.0% | 0.0% |
| Tyr (Y) | 4.3% | 4.4% | 5.9% | 5.5% | 4.9% | 6.3% | 5.2% |
| Val (V) | 1.1% | 4.8% | 3.4% | 0.5% | 4.6% | 1.1% | 2.1% |
| Pyl (O) | 0.0% | 0.0% | 0.0% | 0.0% | 0.0% | 0.0% | 0.0% |
| Sec (U) | 0.0% | 0.0% | 0.0% | 0.0% | 0.0% | 0.0% | 0.0% |
